# Telehealth Autism Diagnostic Assessments With Children, Young People, and Adults: Qualitative Interview Study With England-Wide Multidisciplinary Health Professionals

**DOI:** 10.2196/37901

**Published:** 2022-07-20

**Authors:** Debbie Spain, Gavin R Stewart, David Mason, Victoria Milner, Bryony Fairhurst, Janine Robinson, Nicola Gillan, Ian Ensum, Eloise Stark, Francesca Happe

**Affiliations:** 1 Institute of Psychiatry, Psychology & Neuroscience King's College London London United Kingdom; 2 The National Psychology Clinic London United Kingdom; 3 Berrywood Hospital Northampton Healthcare National Health Service Foundation Trust Northampton United Kingdom; 4 Cambridge Lifespan Autism Spectrum Service Cambridgeshire and Peterborough National Health Service Foundation Trust Cambridge United Kingdom; 5 Bristol Autism Spectrum Service Avon and Wiltshire Mental Health Partnership National Health Service Trust Bristol United Kingdom; 6 University of Oxford Oxford United Kingdom

**Keywords:** autism, COVID-19 pandemic, autism diagnostic assessment, telehealth, health professionals, clinical supervision, training, COVID-19

## Abstract

**Background:**

Autism spectrum disorder (hereafter, autism) is a common neurodevelopmental condition. Core traits can range from subtle to severe and fluctuate depending on context. Individuals can present for diagnostic assessments during childhood or adulthood. However, waiting times for assessment are typically lengthy, and many individuals wait months or even years to be seen. Traditionally, there has been a lack of standardization between services regarding how many and which multidisciplinary health professionals are involved in the assessment and the methods (diagnostic tools) that are used. The COVID-19 pandemic has affected routine service provision because of stay-at-home mandates and social distancing guidelines. Autism diagnostic services have had to adapt, such as by switching from conducting assessments in person to doing these fully via telehealth (defined as the use of remote technologies for the provision of health care) or using blended in-person or telehealth methods.

**Objective:**

This study explored health professionals’ experiences of and perspectives about conducting telehealth autism diagnostic assessments, including barriers and facilitators to this, during the COVID-19 pandemic; potential telehealth training and supervision needs of health professionals; how the quality and effectiveness of telehealth autism diagnostic services can be enhanced; and experiences of delivering postdiagnostic support remotely.

**Methods:**

A total of 45 health professionals, working in varied settings across England, participated in one-off, in-depth semistructured qualitative interviews. These were conducted via videoconferencing or telephone. Altogether, participants represented 7 professional disciplines (psychiatry, medicine, psychology, speech and language therapy, occupational therapy, nursing, and social work). The data were then analyzed thematically.

**Results:**

Thematic analysis indicated the following 7 themes: practicalities of telehealth, telehealth autism diagnostic assessments, diagnostic conclusions, clinical considerations, postdiagnostic support, future ways of working, and health professionals’ experiences and needs. Overall, telehealth autism diagnostic assessments were deemed by many participants to be convenient, flexible, and efficient for some patients, families, and health professionals. However, not all patients could be assessed in this way, for example, because of digital poverty, complex clinical presentation, or concerns about risk and safeguarding. Working remotely encouraged innovation, including the development of novel assessment measures. However, some participants expressed significant concerns about the validity and reliability of remotely assessing social communication conditions.

**Conclusions:**

A shift to telehealth meant that autism diagnostic services remained operational during the COVID-19 pandemic. However, this method of working has potentially affected the parity of service, with people presenting with clinical complexity having to potentially wait longer to be seen or given a diagnostic opinion. There is also a lack of standardization in the provision of services. Further research should identify evidence-based ways of enhancing the timeliness, accessibility, and robustness of the autism diagnostic pathway, as well as the validity and reliability of telehealth methods.

## Introduction

### Background

Autism spectrum disorder (henceforth, autism) is a lifelong neurodevelopmental condition affecting 1% to 2% of the population [1]. Core autism traits include social communication differences (impairments), difficulties tolerating change and uncertainty, sensory sensitivities, and restricted or repetitive interests and behaviors [2]. Autism is a substantially heterogeneous condition. Traits may be subtle or severe, affecting functioning to varying degrees [2]. Some individuals are diagnosed early in life, for example, when parents or teachers notice difficulties. Conversely, many individuals are only seen for diagnostic assessment in adulthood, commonly but not exclusively at the point they are required to become more independent and autonomous [3]. There is also growing recognition that many autistic individuals are undiagnosed or remain misdiagnosed [4].

Autism diagnostic assessments have traditionally lacked standardization between services and settings. For example, data on clinical practice in the United States, Canada, New Zealand, and the United Kingdom indicate that there is variation in how many and which health professionals are involved in the diagnostic process, the semistructured or structured diagnostic tools that are used, from whom information is obtained apart from the person (eg, family and educators), and the topics that the person is asked about (eg, a sole focus on autism or wider themes that include mental health) [5-7]. This is important as the assessment process can influence outcomes (ie, what diagnostic conclusions are reached) [8] and, in turn, the service provision that patients and their families can access.

In England, health professionals are expected to follow the National Institute for Health and Care Excellence guidelines pertaining to autism diagnostic assessment [9,10]. Traditionally, assessments have been conducted in person, with very limited use of telehealth (defined as the use of remote technologies, including videoconferencing and the telephone, for the provision of health care). Irrespective of age, the National Institute for Health and Care Excellence recommends that the assessment comprises a minimum of three components: (1) a clinical interview or assessment with the person, (2) behavioral observation, and (3) a review of developmental history. Although the guidelines provide an indication of the types of semistructured or structured tools that may be useful, they do not mandate the use of one over the other, resulting in differences in practice [9,10].

The emergence of the COVID-19 pandemic in early 2020 substantially affected the provision of emergency, routine, and specialist clinical services. Stay-at-home mandates and social distancing measures meant that nonemergency services needed to adapt the standard ways of working [11,12]. Some autism diagnostic services temporarily shut down waiting lists and suspended direct clinical work for several months, exacerbating already lengthy waiting times [13,14]. However, overall, many services started conducting partial or complete autism diagnostic assessments using telehealth [13-16].

There is a precedent for conducting autism diagnostic assessments remotely [17,18]. For example, 10 studies conducted in the United States before 2020 examined the feasibility and acceptability of conducting assessments via telehealth rather than in person, with preliminary evidence of effectiveness and good interrater reliability (when comparing both methods) [17]. However, the pandemic context, including stay-at-home mandates and social distancing measures, has introduced additional complexities and considerations for clinical practice, such as the need to rapidly develop new systems and processes to facilitate telehealth appointments, the expectation that professionals will adopt new ways of working without formal training, and the need to make clinical decisions about eligibility or contraindications for telehealth in a clinical rather than a research sample. A recently published systematic review of studies investigating telehealth methods of autism assessment and interventions for autistic individuals, conducted before and during the pandemic, also found that this is feasible, effective, and reliable [18].

Since the start of the COVID-19 pandemic, a handful of studies, primarily conducted in the United States and Canada, have examined the feasibility and acceptability of telehealth autism diagnostic assessments or the perspectives of patients or health professionals [14-16,19-21]. Preliminary findings indicate that some professionals and services can find telehealth to be convenient, flexible, and satisfactory when working with individuals across the life span. However, consistent concerns have also been raised by some professionals, including difficulties with engaging patients and families, assessing subtleties in nonverbal social communication and performing risk assessment, limited confidence in reaching diagnostic conclusions, and wider challenges such as technological problems (digital poverty and poor internet connection) and environmental considerations (eg, lack of privacy during appointments). In addition, professionals have identified a paucity of diagnostic tools validated for use via telehealth [22] and more general uncertainty about the validity and reliability of remotely assessing a condition underpinned by social communication differences. Some have also noted the potential for a widening gulf in health care disparities, as factors such as digital poverty, clinical complexity, risk, and the need for interpreters may mean that services want to meet patients and their families in person, resulting in a longer waiting time for assessment. Taken together, the evidence to date suggests that telehealth has merit as an approach for assessing autism [17,18]. However, professionals also face challenges in practice as a direct result of this approach, which may directly affect confidence and clarity in reaching diagnostic conclusions and the resultant service provision available for patients and their families. Further investigation of professionals’ experiences of conducting telehealth autism diagnostic assessments in other settings and contexts is warranted to better understand how service provision can be more suitably tailored during and beyond the COVID-19 pandemic. Moreover, understanding more about barriers and facilitators to telehealth autism diagnostic assessment may help inform future iterations of service provision, ideally incorporating input from the range of involved stakeholders (including patients and their families).

### Study Aims

This study aimed to investigate England-wide multidisciplinary team (MDT) health professionals’ experiences of and perspectives about (1) conducting telehealth autism diagnostic assessments, including barriers and facilitators; (2) potential training and supervision needs of health professionals using telehealth; (3) how quality and effectiveness of telehealth autism diagnostic services can be enhanced; and (4) experiences of delivering postdiagnostic support.

## Methods

We report the methods and findings based on the Consolidated Criteria for Reporting Qualitative Research guidelines [23] (Multimedia Appendix 1 [23]).

### Research Team

The team included autistic and nonautistic researchers working clinically (primarily in adult autism services or mental health settings) or in autism research departments. All members of the team were invited to comment on the study design and methods, as well as interpret the findings and contribute to the write-up. Members of the autism community (autistic teenagers, adults, and a parent carer) were asked to comment on the study materials and findings and offer their perspectives on the implications arising from the research.

### Study Design

This study was informed by phenomenological principles and used a qualitative study design. MDT health professionals attended one-off semistructured interviews between March and June 2021.

### Ethics Approval

Ethics approval was obtained from King’s College London (Research Ethics Committee reference MRA-20/21-22168). The participants provided informed consent, including for the dissemination of anonymized quotes.

### Participants

Participant inclusion criteria were MDT health professionals (eg, representing psychiatry and psychology) with experience in conducting autism diagnostic assessments or providing postdiagnostic support for children, adolescents, or adults in any setting and in England.

We used convenience and snowball recruitment methods via the authors’ existing England-wide collaborations and networks, gatekeepers at health organizations and universities, word of mouth, and social media. Recruitment ceased once (1) the breadth of health professional disciplines involved in autism diagnostic assessments in England was represented and (2) data saturation (defined as no new themes emerging) was reached.

**Figure 1:**
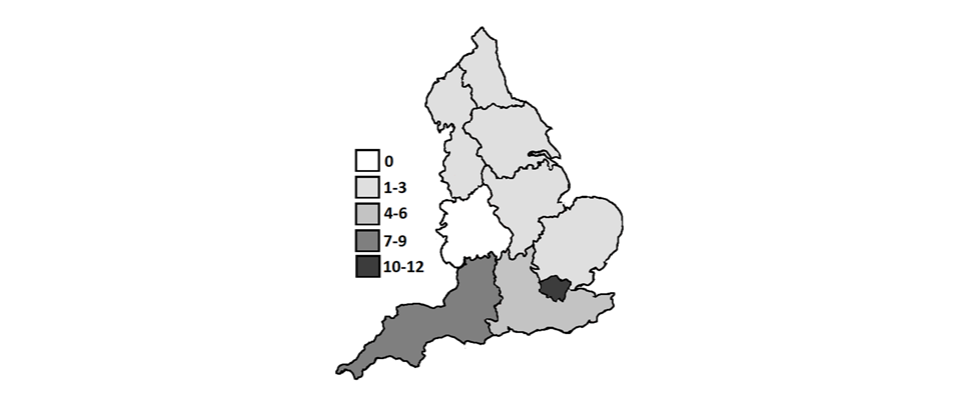
Location of services within which participants were based.

A total of 45 MDT health professionals from across England participated (Figure 1). This comprised most of the total number of potential participants who initially expressed interest in the study. A total of 7 professional disciplines were represented. Most participants worked at least part-time in the National Health Service (NHS). Their expertise in working with autistic individuals ranged from 6 months to 30 years (mean 12 years; Table 1).

**Table 1:** Participants’ professional demographic characteristics (N=45).

Characteristics		Participants
**Profession^a^, n (%)**		
	Clinical psychologist	13 (29)
	Speech and language therapist	6 (13)
	Occupational therapist	6 (13)
	Psychiatrist	5 (11)
	Neurodevelopmental worker^b^	5 (11)
	Social worker	3 (6)
	Pediatrician	2 (5)
	Nurse	2 (4)
	Medical physician	2 (4)
	Counseling psychologist	1 (2)
**Experience (years), mean (SD); range**		
	Since core or primary professional training (n=29)	13.32 (7.11); 1-23
	Working with autistic individuals	12.14 (8.53); 0.5-30
**Service context, n (%)**		
	NHS^c^	32 (70)
	Private	13 (30)
	Accepts referrals for people with a learning disability	8 (17)
	Digital health provider	5 (11)
**Age of patient group (n=35), n (%)**		
	Child (<18 years)	13 (37)
	Adult (≥18 years)	17 (49)
	Life span	5 (14)

^a^Participants could endorse >1 professional discipline.

^b^Unqualified practitioner specializing in administering semistructured diagnostic tools as part of the multidisciplinary team assessment.

^c^NHS: National Health Service.

### Materials

The topic guide informed the interviews. This was developed in collaboration with experts with experience and health professionals. Briefly, the topic guide included (1) demographic questions, (2) contextual questions about participants’ service context and experience of using telehealth or hybrid assessment, (3) prompts about views on telehealth, (4) perceived telehealth training and clinical supervision needs, and (5) thoughts about improving service provision during and beyond the COVID-19 pandemic. Multimedia Appendix 1 provides more information regarding the topic guide.

### Procedure

Interviews were conducted by 3 female researchers (DS, BF, and VM)—1 postdoctoral nurse, 1 clinical psychologist, and 1 doctoral student—all of whom had experience in conducting qualitative research and autism diagnostic assessments in varied settings (including inpatient and community settings, the criminal justice system, and research studies). Interviews were conducted via videoconferencing, as well as, infrequently, by telephone, at the time of participants’ choice. Of the 45 participants, 6 (13%) had prior working relationships with their interviewers. This was acknowledged but not considered to impede participants’ responses, as in all but one instance, individuals were not routinely working together at the time of study participation. The participants were aware that the study focused on the clinical practice and research interests of the team.

During the interviews, participants were asked questions based on topic guide prompts, allowing them to lead the conversation. The mean duration of the interviews was 46 (range 20-73) minutes. Interviews were recorded (audio and video), excluding 2 instances because of technical issues. The participants were not asked to specify their location at the time of the interview, although most appeared to be at work or home. We did not ask whether there were others in the vicinity during study participation; however, there were no obvious interruptions. Interviewers took hand notes and met intermittently during the study setup and recruitment for peer reflection on the interview process and content and to ensure that they each met participants with different backgrounds (ie, working with young people or adults, in the NHS or independently, and from varied professional disciplines).

### Data Analysis

Data pertaining to participants’ professional demographic characteristics and descriptions of service-related factors were summarized descriptively. Qualitative data were analyzed thematically, involving (1) becoming familiar with the data, (2) generating initial codes, (3) searching for themes, (4) reviewing tentative themes, (5) labeling themes, and (6) summarizing the data [24].

Reflexivity was embedded in the research process and during the thematic analysis [25]. This included members of the research team reflecting on perceptions of how autism can and should be assessed; minimum standards for autism assessment; views about the utility, validity, and reliability of telehealth; and experiences of receiving or providing autism and nonautism diagnoses in person and remotely.

The interviewers transcribed their own interviews. Transcripts were not sent to the participants for comments or checking. These were collated into a master document organized according to the question topic. One of the researchers (DS) created initial codes that were subsequently refined through categorization, with labels assigned to tentative themes and subthemes as they were identified. To ensure consistency within the coding, 2 researchers (VM and BF) randomly selected 10% of the interview responses at random and coded them. The codes were then compared among all 3 interviewers, with a high degree of comparability. Tentative themes were then finalized and presented to the wider research team for their comments.

## Results

**Figure 2:**
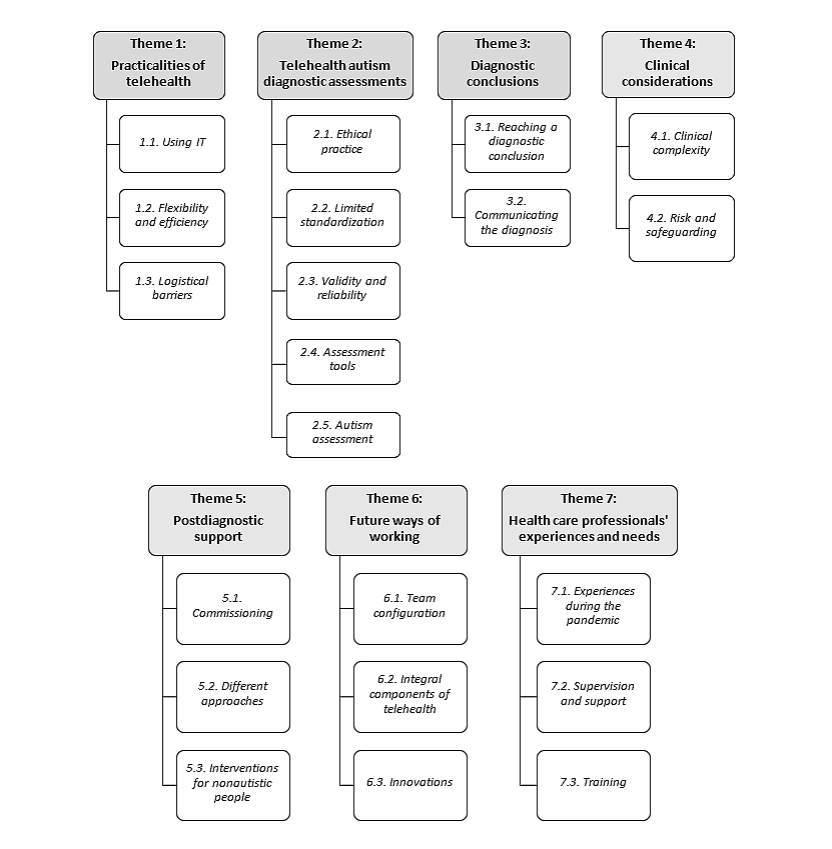
Overview of themes and subthemes.

Thematic analysis of the data indicated there were seven themes (1) practicalities of telehealth, (2) telehealth autism diagnostic assessments, (3) diagnostic conclusions, (4) clinical considerations, (5) postdiagnostic support, (6) future ways of working, and (7) health professionals’ experiences and needs. Figure 2 presents the themes and subthemes (Multimedia Appendices 2-8).

### Theme 1: Practicalities of Telehealth

The first theme pertained to participants’ views on the practicalities of using telehealth, with three subthemes: (1) using IT, (2) flexibility and efficiency, and (3) logistical barriers.

#### Using IT

*Digital poverty* was an issue for some participants. One of the participants said the following:

...privileged people can access a lot better and get a much more robust kind of assessment, because it’s not constantly losing connection all the time. So that’s a real concern to me.

Professionals working in teams were said to have differing *computer literacy levels* and access to technology or devices. Some patients and families were described as being accustomed to IT, whereas others could *struggle with technology*. The lack of familiarity with this could be *an independent source of anxiety*. One participant remarked they “have one laptop to read, write and call from...the IT is not enough...we need bigger [wider], and more screens,” specifically, one to use for clinical interactions and a second for reading and writing notes.

The use of videoconferencing platforms also differs. For example, Zoom videoconference was permitted only in some NHS Health Trusts. One of the digital health services used a custom-built platform. Some participants noted complexities associated with not being able to blur the screen background:

[this] means that people might know more about your personal life than any of us might share

The visible contrast between some participants’ and patients’ home environments could be stark:

...there might be things in the background that are a bit distracting for somebody

These factors may have influenced engagement and rapport building between patients and professionals, as well as patients’ attention during an assessment.

#### Flexibility and Efficiency

The consensus was that telehealth “gives us flexibility and choices.” Together, participants said that using telehealth could result in (1) fluid appointment times, (2) more accessible appointments, (3) fewer no-shows, (4) options for swiftly filling last-minute cancellations, (5) the possibility for audio and video recording of assessments, (6) less travel and minimal expenditure (eg, on travel or parking), (7) fewer room bookings, (8) environmental benefits or lower carbon footprint, and (9) capacity for recruiting staff living outside of the area. Consequently, many participants felt that flexibility in telehealth could benefit all stakeholders.

#### Logistical Barriers

Participants described a range of logistical issues related to patient circumstances that could potentially influence the viability, practical implementation, and success of a telehealth autism assessment (Textbox 1).

**Logistical issues affecting telehealth autism diagnostic assessments.**

- Environmental factors
  Location not optimal for an assessment (eg, nowhere suitable to sit at home, dialing into the appointment from work, and walking or riding a bicycle while doing the assessment)
  Domestic situations not optimal for an assessment (eg, lack of privacy and caring for young children during the assessment)
  Poor lighting or curtains closed
  Poor sound or much background noise
  Distracted by extraneous cues, finding it hard to sustain attention, and experiencing difficulty in sitting still
- IT-related factors
  Issues relating to the camera (eg, height and position of the camera, proximity to the patient, and declining to turn the camera on or camera turned off unexpectedly)
  Battery of device running out of charge unexpectedly
  Only possible to see what is in front of the camera and not behind or at the periphery
- Engagement-related factors
  Can feel intrusive to speak to someone while at home
  Patient may choose not to join the assessment or wander off part of the way through
  Displaying behavior that seems inappropriate for the context (eg, patient or family members not fully clothed, disappearing to make a sandwich or go for a walk, and answering the telephone)
- Risk-related factors
  Domestic abuse
  Safeguarding issues

### Theme 2: Telehealth Autism Diagnostic Assessments

The second theme pertained to the views of the participants when using telehealth for autism diagnostic assessments, with five subthemes related to (1) ethical practice, (2) limited standardization, (3) validity and reliability, (4) assessment tools, and (5) autism assessment.

#### Ethical Considerations

Several participants stated that it was unethical for patients to wait longer than necessary. Thus, telehealth was a reasonable option, given the COVID-19 pandemic. Conversely, another participant highlighted that some patients cannot be seen using telehealth (eg, as they do not have IT or their clinical presentation precludes this [see the *Theme 4: Clinical Considerations* section]), and thus, “there’s a bit of an ethical dilemma there, because obviously they’ve lost their place on our waiting list.”

It was also reported that a purely remote assessment may contravene *ethical professional standards*. As autism is a social communication condition, not meeting a patient in person may mean that naturalistic interactions cannot be adequately assessed.

#### Limited Standardization

Autism assessments lacked standardization, as highlighted by one of the participants, who said that “everybody’s making their best guess at what might work.” Services differed in terms of the (1) number of health professionals involved, (2) depth of information obtained, (3) range of sources from which information was gleaned, (4) types of behavioral observation assessments used, (5) setup of in-person appointments when offered, (6) total number of appointments offered (including feedback), and (7) overall duration of the assessment.

#### Validity and Reliability

Participants’ views differed regarding whether this method of diagnostic assessment was valid and reliable. Reflecting the views of many, as well as a change in usual practice since March 2020, one of the participants said the following:

I’ve been really surprised as how useful it is...[before the pandemic], I thought it would be a really bad idea and it wouldn’t be valid, and it would be very limited, not reliable...now I’ve really shifted.

However, this was commonly caveated with *curiosity* and, more specifically, a worry, about “how valid and reliable it is,” especially the behavioral observation components of the assessment (see the *Assessment Tools* subsection). Conversely, others said the following:

...feel so strongly about it that it’s not valid...if the full assessment is done remotely, it’s not clinically valid

I couldn’t in all conscience assign a diagnosis [about] something as profound as how you interact socially with another human being having never sat in a room with them.

Some participants thought telehealth assessments “do work very well, but there are always going to be [patients] when they’re not going to be sufficient.”

The reliability of telehealth assessments could be dependent on the age of the patient, such as being less appropriate for younger children. Others have suggested that this is less reliable for people with “definite speech and language difficulties, with intellectual [learning] disability, learning difficulties such as dyslexia, dyspraxia,” or parents with a learning disability, who may find this a more overwhelming, ambiguous, or confusing meeting.

#### Assessment Tools

Obtaining a developmental history, such as with the Autism Diagnostic Interview–Revised [26], was considered easy via telehealth, and indeed, this commonly occurred before the COVID-19 pandemic. Formal behavioral observation assessments, such as the Autism Diagnostic Observation Schedule (ADOS)–2 [27], translated less well to web-based forums. Some services decided to complete the ADOS-2 when social distancing measures were no longer in place, resulting in patients being placed on an internal waiting list.

Many services demonstrated innovation and developed an *ADOS-informed assessment*, comprising play-based and conversational tasks. Participants found this beneficial for structuring an appraisal of behaviors suggestive of autism. However, it was noted that these assessments had not been empirically tested, and thus, their psychometric properties (eg, test-retest reliability and interrater reliability) were unknown. On reflection, one of the participants felt that the ADOS-informed assessment they were using “is slightly limiting. We’ve done the best we can.” Another said they had adopted “a really low threshold for review when we weren’t certain...we feel that we may be missing things.” Others described their newly developed assessment as “really successful, and I think it’s been amazing that there’s certain things you can pick up doing it.”

#### Autism Assessment

Opinions on conducting telehealth autism assessments varied. Some participants said that, with practice and experience, this did not differ substantially from assessing someone in person:

...we’ve certainly adjusted to it and for a significant majority of people, doing online assessment has been absolutely fine, and I think the diagnostic conclusion we’ve made has been the same as to whether we’d seen them in a room or not

Another suggested the following:

...a difficult case is a difficult case, and a straightforward case is a straightforward case...I’m not sure that meeting somebody in person would have made a big difference

Some characteristics prototypically associated with autism could be challenging to observe via telehealth, summarized as “you lose a lot of the subtleties...lose out on the interaction.” These included (1) nonverbal behavior (eg, eye contact and quality, flexibility, range, congruence, and integration of facial expressions and gestures); (2) fluidity, responsivity, and reciprocity of social interaction with familiar and unfamiliar others; (3) hypo- and hypersensory sensitivities (eg, to light or noise); (4) repetitive movements and mannerisms, especially those outside the camera shot; and (5) gait and posture.

In addition, it could be difficult to assess coping strategies patients use in their day-to-day lives to manage difficulties or traits:

...you might not see that the curtains are drawn, or you might not see that there’s particular lighting that they need

The medium of telehealth could affect judgment about why a trait or behavior was observed. Echoing others’ comments, one of the participants said the following:

...how much of that [social interaction difficulties] is a deficit on their part, and how much of it is just because there might be a slight delay in the internet? Or there might have been a break in the connection. So, it can be complicated to figure out whether their difficulty with reciprocity is because of that, or whether it’s a typical issue.

With younger children, there was a specific concern because of the following:

...they’ve not really had much social interaction over the last year [2020], and then you’re trying to discern whether that’s a COVID thing, or whether that’s related to how they prefer things to be anyway

Seeing patients’ home environments could help with finding out about their preferences and difficulties:

I like that the person can show you things in their home. So, if you’re asking somebody about collections, they can then show you that collection, or if they if you’re asking about organisation, you know they can show you things that they’ve organised and so you get that sort of evidence and insight that you wouldn’t get by bringing somebody to a clinic.”

It was also easier to “see family dynamics” and “parent child interactions...like mum putting a hand on the child’s shoulder...little things that actually show you a little bit about what their relationship is like.” In contrast, patients could access their “favorite toys” at home; more easily rely on a “scripted, rehearsed kind of story”; and thereby, manage some interactions and ADOS-informed tasks more adeptly.

The remote assessment of domains other than autism could pose challenges. Several participants noted that there is “no possibility of doing a physical examination, not even just blood pressure and pulse, or you know if you thought someone would benefit from blood tests.” Alongside this, many participants described difficulties in assessing the nonspecific elements of social interaction informing diagnostic conclusions:

...do they hold the door open for the informant, then let it go in their face

...who sits next to who

...how do they greet me...how do they sit

...how they cope by coming to a clinic

...if someone’s trousers are stained, or if someone smells, like you’re not getting that information about self-care and things that they might be struggling with and they don’t always have the insight to be able to give you that information verbally

Some participants said it was more difficult to develop a rapport on the web:

[it’s] nice and tangible sitting in a room and there’s some natural toys, and let’s do this task together and let’s work on it together

A few participants wondered the following:

...people find it easier to sort of spin you a mistruth, when people want a diagnosis...I’ve got a lady at the moment that I don’t know if she has autism or not, but she’s giving me a lot of conflicting information

### Theme 3: Diagnostic Conclusions

The third theme pertained to formulating and sharing diagnostic conclusions with two subthemes: (1) reaching a diagnostic conclusion and (2) communicating the diagnosis.

#### Reaching a Diagnostic Conclusion

As for reaching a diagnostic conclusion, one of the participants said the following:

I think a lot of it’s to do with the experience. The more you see people with different types of ASD and different presentations, and it takes a long time, but you find patterns in things and in people’s behaviours, so you know you can read [about] it as much as you want or go on as much training courses that you can, but you never quite get it until you’ve been working with individuals for a long period of time

Others felt that telehealth assessments introduced greater “uncertainty...we spend longer discussing cases,” and the following:

I think it [telehealth] makes it much harder as a clinician to be sure of the diagnosis...you can’t rely on your feeling and your responses because you’re just listening to what they’re saying

There was a sense of *complexity* and difficult diagnostic decisions, and more difficulties for *newly qualified* health professionals. Several participants said they would not “confirm a diagnosis with anyone that I have not seen in person.”

A few services had adopted an open-door policy, with one participant describing the following:

...assessment is limited by the set up [telehealth]...we generally say we would be happy to review in two years if problems persist. So, if we haven’t given a diagnosis, we’re leaving the door open that we may have missed it

Moreover, their service “made recommendations based on the young person’s current needs and situations, so we might give autism-related recommendations even without the diagnosis.”

#### Communicating the Diagnosis

In some services, feedback was provided in person, resulting in a lag between assessment and diagnosis. Some participants felt the following:

...quite callous and not particularly warm and friendly to be doing it over [the internet], like you’re giving someone a life changing diagnosis and you can’t even offer them a cup of tea while you’re doing it or something. You know there’s nothing to kind of soften the blow

Another said the following:

...it’s difficult if they are very emotional—you can say warm empathic things, but you can’t hand them the tissue box...you feel a bit inadequate

Some noted that giving a diagnosis jointly with colleagues seemed easier than giving a diagnosis just as the sole health professional.

The patients’ experiences of receiving a diagnosis were important. It was difficult to know “whether it feels better for them to be in their own space and try to process that, or whether it’s better to be in a clinic room.” Some patients were said to “underestimate what the impact of a diagnosis might be like for them.” One participant highlighted the following:

...there’s a bit [of a] difference about you sitting in your bedroom and somebody giving you some news and then hanging up and you’re still kind of sitting in your bedroom, versus coming to a room, somebody telling you something, you’ve been given kind of that time in the room, and then leaving the place where you’ll be given the outcome to travel somewhere different

Others said receiving a diagnosis could be a relief for patients; however, this was communicated, such as “it explains my past. You know, I’ve got a different narrative now.” Overall, ensuring that patients have “the right emotional support around them” was deemed crucial.

Not receiving an autism diagnosis could incur *frustration and sadness*. Participants reported that relaying this in person or via telehealth could be difficult. One of the participants said their service goes “that extra mile” if a patient does not receive a diagnosis, as “you’ve got to do that in a way that doesn’t [seem] over rejecting...like a huge disappointment.” A few participants dealt with formal complaints whereby parents had said that a diagnosis of autism was not made as the assessment had been conducted via telehealth.

### Theme 4: Clinical Considerations

The fourth theme pertained to clinical considerations associated with the feasibility of using telehealth in their service, with two subthemes: (1) clinical complexity and (2) risk and safeguarding.

#### Clinical Complexity

Participants said that referrals were increasingly being received for “more and more complex cases.” Examples of complex cases may include patients presenting with limited verbal communication or selective mutism, mental health conditions, enduring personality traits or personality disorders, attachment-based problems, complex trauma, *looked-after* child status, fetal alcohol syndrome, sensory processing disorders, multimorbidity, or a forensic history. Some participants felt that the COVID-19 pandemic resulted in *a 2-tiered system*, with patients with more straightforward presentations being seen via telehealth versus patients with more complexity possibly waiting for longer.

Clinical complexity typically meant that the assessment was “more of a challenge”:

...because we need to have more discussion and the MDT process becomes more lengthy, because you have got more to consider

Participants found this could make it “really hard to tell whether they’re [patients] autistic or not autistic, and you go away kind of thinking, well after 10 years, I should be able to know whether someone’s autistic or not. It’s very rare that I can’t reach a conclusion [in person], but it seems to be far more complicated [via telehealth].”

#### Risk and Safeguarding

In some services, moderate to high risk to self or others, recent suicidality, substance use, *high mental health needs*, impaired capacity, and known safeguarding concerns precluded the offer of a telehealth assessment.

Participants identified a range of risks inherent in clinical work, including to self, to others, and from others. However, the current pandemic context potentially increased the risk for some people, such as “from the fact that you’re doing [the] assessment remotely.” For instance, the following was more crucial:

...know where somebody is when you’re speaking to them [as] they may not be at home...if there were kind of risk issues that came up, it will be important to know where they were.

Another participant said that *risky topics* could arise when someone “doesn’t want to disclose the ASD assessment to their family or partner or their children.” Providing feedback for a diagnostic conclusion that patients are not happy with could also feel risky, especially in the absence of good rapport developed in person. Several participants highlighted that there may be an increased “risk of getting it [the diagnosis] wrong” and incurring “false positives and false negatives.”

Several participants expressed uncertainty about whether the risk can be accurately gauged remotely, with some feeling “it can be quite difficult to hold that risk remotely.” This was deemed especially tricky, as “there isn’t anyone else that’s going to come and pick up and monitor that risk.” Another said the following:

...assessing high risk patients...[such as]...someone who’s very psychotic... creates a bit more anxiety rather than being with the person in the same room and kind of getting a sense of the situation.”

Alongside this, it was noted that risk assessment and management is core work for some professional disciplines (eg, psychiatry, clinical psychology, and nursing); however, there may be less emphasis on this in the training of other disciplines:

...there’s extra training to try and bring everybody up to that standard, which is really good, but then sometimes there are still gaps in people’s knowledge and experience

It was also apparent that a few services declined referrals for patients deemed to present any risk, again highlighting the potential disparities.

Seeing patients in their homes, via the web, could raise unexpected safeguarding concerns. One of the participants said the following regarding the period of a break:

...parents forgot to turn off the camera and volume, and they [the professional] heard inappropriate things where they shouted at their children...it made them feel uncomfortable and they filed a safeguarding concern

Another participant identified that talking about safeguarding could potentially increase the risk of further safeguarding issues; for example, when assessing someone in an “abusive...coercive relationship” seen in the company of the abuser.

Participants talked about the complexity of dealing with safeguarding issues from their own homes:

...there’s just something about being in a clinic environment where you know you almost kind of have your like safeguarding hat on more. I think because you’re kind of in a role, whereas when you’re at home, sometimes say you know you hear something or you even see something in the background, and I get a moment where I think gosh, this is actually really, you know important...sometimes that’s difficult and not having just that constant kind of liaison with your colleagues is really hard

### Theme 5: Postdiagnostic Support

The fifth theme pertained to the participants’ views on postdiagnostic support and how their service currently handles this, with three subthemes: (1) commissioning, (2) different approaches, and (3) interventions for nonautistic individuals.

#### Commissioning

Many services were “not commissioned to provide any postdiagnostic support,” although this was described as follows:

...crucial, because we’ve got lots and lots and lots of children and adults who are being diagnosed with autism. But then, [they ask], what now? Where do I go with this? How can I make this useful?

Another participant emphasized the following:

...they [commissioners, managers] sometimes lack the understanding that it is much more than a diagnosis or not. It’s about being able to come away knowing that you feel that you’ve got a pretty good understanding of that child to not only feel comfortable making the diagnostic decision that you made, but also that you’ve been able to do something helpful for families

The consensus was that services should be better resourced to provide input after the assessment.

#### Different Approaches

The nature of postdiagnostic interventions differed between services, ranging from no intervention; signposting; resource leaflets; in-depth assessments of functioning; psychoeducation workshops and groups for patients, families, or friends; regular drop-in sessions; and, infrequently, individual sessions.

Some services had moved groups to the web, with varying degrees of success. One of the participants described their group now “feels much more like a teaching session...most of the clients don’t want the camera on… so you can feel you could be speaking into the empty [void].” Others considered the move to web-based groups to have “been more successful than I thought it would be”—a valuable asset for patients who may have opted out of or been unable to attend in person. Attending the group on the web also meant that “you don’t have to talk, but you can listen,” reducing potential pressure on patients.

#### Interventions for Nonautistic Individuals

The lack of a postdiagnostic intervention for people who do not receive a diagnosis of autism was mentioned:

...if you don’t have a diagnosis of autism...this is a big issue. Too many autism services just dump them

In one of the services, importance was placed on parity of understanding irrespective of diagnosis:

...you still get all of that same process. You still get the formulation. You still get told you will still get a differential diagnosis and opinion and we will still make recommendations for you. So, no matter where you are, autistic or not, you come up with the full assessment and what’s deemed to be your diagnosis, but also what’s deemed to be a formulation, so that if you do have to go into other services, you can take that with you, not have to answer the same questions again

### Theme 6: Future Ways of Working

The sixth theme pertained to participants’ thoughts about optimal service provision, with three subthemes: (1) team configuration, (2) integral components of telehealth, and (3) innovations.

#### Team Configuration

There was wide variation in workforce configurations. Few participants worked as sole practitioners. Most teams had between 2 and ≥6 professional disciplines represented or available to participate in assessments ad hoc.

Echoing many participants’ sentiments, one of the participants said the following:

I don’t think they [health professionals] need to be from a particular professional background. What’s more important is that they have adequate experience and training and confidence in differential diagnosis across a range of mental conditions and a range of neurodevelopmental conditions and that they know the [care] pathways, whether that’s in the private sector or the NHS, you know, to refer people on for follow-up assessments and follow up treatment

#### Integral Components of Telehealth

Of the 45 participants, 5 (11% of the sample) worked for a digital health service. Of the remaining participants, most perceived services will continue to use telehealth beyond the COVID-19 pandemic. Whether this would be augmented with at least one in-person appointment depended on factors such as (1) organizational policy, (2) patient choice, (3) clinical complexity, (4) potential risk and safeguarding issues, (5) health professionals’ preferences, and (6) environmental considerations (eg, whether there is somewhere quiet and confidential that patients and health professionals can use in their own homes or work areas). Reflecting many others’ perspectives, one of the participants noted they are “happy to advocate a hybrid model, as long as the hybrid model is being hybrid to increase capacity without losing quality.”

Participants outlined the fundamental elements they considered necessary to ensure good quality of telehealth autism assessments. This included suggestions for what the assessment comprises, how it is offered, who conducts it, health professionals’ proficiency and ongoing supervision and training needs, and robust processes for service delivery (Textbox 2).

**Fundamental elements of telehealth autism diagnostic assessments.**

- Professional resources
  Remote assessment tools that are evidence based, standardized, and validated
  A computer that has a reliable internet connection
  Robust IT systems, prompt support with IT problems, and clear IT policies
  Excellent admin support and tight admin processes
- Service design and processes
  Collaboration and coproduction of service design and delivery with patients and families
  Blended or hybrid model of service delivery, incorporating remote and in-person options based on needs and preferences
  Differentiated pathways and options for straightforward and complex assessments
  Allocation of patients to a professional within the team to offer continuity from referral to discharge
  Clear procedures for assessing and managing risk or safeguarding concerning and a mechanism for obtaining urgent clinical advice
  Options to conduct a neurodevelopmental assessment
- Team working and supervision
  Input from a range of multidisciplinary team professionals
  Options for joint working with colleagues if clinically indicated
  High-quality clinical supervision
  Opportunities to obtain peer support and build consensus about good practice in telehealth with colleagues at wider services
- Training
  Adequately trained professionals with expertise in autism and mental health
  Guidance on what a good practice telehealth assessment should incorporate and minimum standards for this
- Patient-friendly
  Culturally aware service provision
  Resources for patients (eg, visual information about the assessment process, overview of telehealth etiquette, and computer and internet access)
  Accessible clinical reports and options for patients to comment on a draft report and discuss the final report
  Postdiagnostic support via varied means (eg, written resources, in-depth assessment of functioning, individual sessions, and group support)

#### Innovations

One of the participants highlighted the following:

...how fortuitous it is that COVID’s come along at exactly the time when we’ve got the technological ability to do this stuff

The key to this was the development of new autism assessment tools, taking into account “the cultural differences, and the social cultural context that people are living in.” There was a keenness for “something that does what the ADOS [does], but works in an online environment,” with established validity and reliability.

Potential identified innovations included allowing the patient or parents to forward videos of behavior and functioning in everyday situations, using 2 cameras to observe behavior from different angles in the clinic or at home, developing more eye-tracking or neuropsychological tasks for remote use, and having more sophisticated *screen-sharing* options.

### Theme 7: Health Professionals’ Experiences and Needs

The seventh and final theme pertained to the participants’ experiences and needs as health professionals, with three subthemes: (1) experiences during the COVID-19 pandemic, (2) supervision and support, and (3) training.

#### Experiences During the COVID-19 Pandemic

The convenience, flexibility, and efficiency of working from home were favored by the participants. However, this was not without its limitations. One of the participants said the following:

...all of us are females in our team and [the] majority of us [have] got children as well, so it’s been a bit of a balance really, having time to home school and time to do the assessments

Many participants reported that they “like going to an office and seeing people and being around people” and “prefer sitting in a room with somebody...just to maintain human connection.” Time spent, in person, with colleagues was “absolutely critical...[for] things like humor, team building.”

Several participants had experienced a sense of *isolation*, with one remarking the following:

I’ve never met my team. I’ve never met my supervisor. I’ve never met my patients in person...also it felt very isolated with the team and definitely didn’t help with some team dynamics...sometimes it’s nice to knock on somebody’s door and asking the question, or at least meet the people we work with

Another said the following:

...it’s difficult working with silence...my mental health is not so good, I think, since I’m always on my own

Some general health implications of working at home are highlighted. This could be “more tiring” and “physically intense...I’ve been having eyestrain and more headaches.” Back problems because of “sitting so much” were more common. Another participant said, “the longer [you] spend on a screen, the more burnt out you feel.” Overall, it was suggested that “actually getting up and out of your seat, and not working from a computer all the time, is actually physically more healthy.”

#### Supervision and Support

In keeping with several participants’ viewpoints, one of the participants noted the following:

I think we’ve been making things up as we go along and there hasn’t been very much guidance from anywhere about what we

should do

Supervision was deemed “more important now than ever, but it’s more avoided. I think because people are just so tired with it all [the pandemic].” Some participants expressly wanted “safeguarding supervision.”

Some pandemic-specific reasons for supervision were described, including the following:

...thinking about the impact of us not having our own routines or home life balance being so blurred, and helping people to find ways to separate work and home when they’re in the same environment...the impact of the pandemic on everybody and how it changed everybody’s life...emotional demands [of the] clinical job...we’re all kind of going through you know extreme stress in our lives

Several participants felt that current ways of working raised *ethical considerations* for discussion in supervision:

...holding [the] tension between what do I clinically feel is the right thing to do...what do families want...what is driving the decision-making process?

One of the supervisors reflected they are “a bit more careful when [they’re] supervising remotely and they’ve [the supervisee] assessed remotely,” to ensure the diagnosis reached is accurate. Peer supervision was also described as “really important...[there is] a real power in hearing from other people.”

Forums bringing together health professionals working across services were considered useful, with one participant saying the following:

...it [would] be fantastic to you know, see what other people [health professionals] have done and how people have changed things and what they feel, or even if it’s just to confirm that what we’re doing is as good as we can do

#### Training

The following was highlighted:

...none of us were trained to do electronic-based assessments as part of our background core clinical trainings. We’ve been forced into it. Some people have flourished staff wise, others haven’t

Few patients had received any telehealth-specific training.

Participants identified five telehealth-specific training areas for health professionals: (1) IT skills (eg, general computer literacy, using video conferencing platforms, touch typing, and digital security), (2) clinical skills (eg, knowledge of mental health and differential diagnoses and how to assess them through telehealth, conducting virtual risk assessments and management, and addressing safeguarding concerns remotely), (3) therapeutic skills (eg, deportment on the web, how to enhance virtual engagement, and rapport building), (4) autism-specific skills (eg, how to assess core symptoms and strengths on the web and training in using new [validated] diagnostic tools), and (5) reliability meetings (ie, checking consistency for clinical assessments and standardized tool use).

## Discussion

### Principal Findings

This study gathered the perspectives of professionals working across services in England and with people across the life span regarding their thoughts about and experiences of conducting telehealth autism diagnostic assessments since the start of the COVID-19 pandemic. The participants represented 7 professional disciplines and had varied experiences with autism services.

A thematic analysis of participants’ responses indicated that there are several advantages associated with telehealth, particularly in relation to convenience, flexibility, and efficiency for patients, their families, and professionals and giving rise to opportunities for innovation. However, participants also reported that telehealth incurs a range of challenges, including increasing potential health care disparities; affecting confidence in assessing, formulating, and sharing diagnostic conclusions; and contributing to clinical, environmental, and practical complexities.

### Comparison With Prior Work

The findings reported here are broadly consistent with those outlined in a handful of recent studies examining professionals’ experiences of providing telehealth autism diagnostic assessments in the United States and Australia [13,16,19]. Studies have reported that professionals appreciated the convenience, flexibility, efficiency, and cost and space savings of telehealth. Moreover, many professionals felt this was an acceptable and satisfactory approach during the COVID-19 pandemic, even if they would not have traditionally opted to work in this way—a finding that echoes the broader literature on telehealth [28]. This is encouraging, although further studies are needed to establish why some professionals are more in favor of telehealth methods for autism diagnostic assessments than others; for example, whether contributory factors for this include the amount of autism- and mental health–relevant experience or expertise professionals have, the type of setting they work in, their age (eg, familiarity with IT), and the degree of training and clinical supervision or support provided within services.

Similar to the findings of this study, professionals elsewhere have raised concerns about the validity and reliability of telehealth autism diagnostic assessments and difficulties in assessing core autism traits remotely [13,15-19]. Concerns have likely been amplified by the fact that professionals are unable to use mainstay diagnostic tools, notably the ADOS-2 [27], and they may not yet be trained in alternatives with preliminary validation (eg, the Brief Observation of Symptoms of Autism) [22].

A consistent theme in the emerging literature is that the subtleties of social communication (eg, modulation of eye contact and use of descriptive or emphatic gestures) can be more challenging to assess via videoconferencing; for example, given the relatively small screen and that nonverbal gestures may not be oriented toward the camera, even if directed to the screen. Similarly, repetitive behaviors (eg, mannerisms) may manifest during an assessment but outside of the camera view. In addition, stay-at-home mandates and social distancing measures have meant that many individuals have had less social contact outside their immediate family in the past 2 years than before 2020 [27]. Indirectly, this may have altered the frame of reference for social situations or social norms for young children or individuals who have been more isolated [29]. Conceivably, some individuals may experience heightened social anxiety. Therefore, in some instances, social difficulties may be evident at assessment; however, causal mechanisms (eg, autism, anxiety, and lack of exposure to social situations) may be uncertain. The implication is that professionals may need to spend more time with patients; for example, conducting an assessment over several appointments so that the individual becomes more familiar with the professional and process, speaking to others who know the person well, or clarifying differences in social styles before and during the COVID-19 pandemic. Conducting assessments jointly with a colleague or developing checklists or prompts for quantifying subtle and overt traits associated with autism may prove useful. Although there is tentative evidence of the reliability of telehealth autism diagnostic assessments [17,18], most studies were conducted before the COVID-19 pandemic. Further research is needed to establish the psychometric properties of the newly developed diagnostic tools and ways through which the validity of telehealth can be enhanced.

Importantly, digital poverty was highlighted as a potential contributory factor increasing health care disparities in this study (ie, not all patients waiting for an autism diagnostic assessment could be seen as they did not have the requisite IT equipment or reliable internet access). This reflects findings reported in studies of telehealth autism services [16,19,29] and telehealth health services more generally [30,31]. Digital poverty is not uncommon [31-33]. For example, approximately 1 in 10 United Kingdom households does not have access to a PC or mobile device with interactive access [31]. For individuals who do have access to an internet-enabled device, it may be that practicalities or cost render internet access difficult, or it may be that they lack the skills or confidence to use this adeptly [32]. Although internet use has broadly increased over the past decade (from 79.7% to 90% of the United Kingdom adult population), it is a cause for concern that individuals with longer-term health issues or from lower socioeconomic status backgrounds may be excluded from telehealth opportunities, or lack access to skill-based training or support to use this. In addition, poorer than required computer literacy of patients, their families, and professionals was highlighted as an important consideration in this study, mirroring findings elsewhere [13,16]. The unexpected onset of the COVID-19 pandemic is likely to have meant that some services were unable to swiftly assess digital poverty and competencies of patients, families, and professionals and accommodate needs accordingly. However, going forward, it is imperative that these factors are addressed with future implementation of telehealth policies, ensuring that patients and families, including individuals with neurodevelopmental or intellectual disabilities [34], are supported to access and use telehealth with ease and that professionals have the correct training and tools to conduct high-quality assessments [35]. Moreover, it would be ideal for all stakeholders to input into co-designing telehealth methods and platforms [34]. This may also include identifying which methods of telehealth are deemed more satisfactory by patients, their families, and professionals; why this is the case; and how this can be used to further iterate the services provided.

Participants identified several fundamental aspects that they considered pivotal for enhancing telehealth autism diagnostic assessments during and after the COVID-19 pandemic. These related to iterating service provision in collaboration with patients and families, offering blended models of care (ie, in person and telehealth), streamlining administrative and IT processes, ensuring patients and families have access to resources, providing professionals with the necessary equipment and training, improving team cohesion, and providing professionals with adequate clinical supervision and sources of support locally and nationally.

Recent studies [16,36,37] have similarly reported that systemic changes to service provision may enhance telehealth in autism services, as well as the acceptability and satisfaction of patients, their families, and professionals. At the onset of the COVID-19 pandemic, many services were able to maintain routine care. There was likely limited time to stop and think, broadly and systemically, about what processes might be best and why. In addition, it was not clear how long the service provision would be disrupted. Now that there is more clarity and possibly more stability in light of vaccination programs, it would be useful for clinical services to evaluate and audit provision and practice during the past 2 years.

Participants in this study identified telehealth-related areas for continuing professional development, including general clinical (eg, engagement), autism-specific, and practical skills. Studies conducted with professionals using telehealth for autism diagnostic assessment [13,16,19] or interventions for autistic individuals [26] have similarly highlighted additional training needs arising in this context. Although it is understandable that services may not have been geared up to offer specialized training at the onset of the COVID-19 pandemic, 2 years in, it seems crucial that core professional training and postqualification training incorporate skills-based sessions to support professionals in developing their competence and confidence in using telehealth. Future research could examine the impact of training on clinical work and whether the mode of delivery (eg, in person vs lectures on the web vs simulation methods) is a moderating mechanism. Ultimately, professionals are likely to require skills that enable them to relatively adeptly use blended in-person or telehealth methods.

### Limitations

This study had several limitations. We recruited participants from a wide sampling frame but were unable to assess the reach of the study information (ie, the number of potential participants who saw the study information vs the number who contacted the research team to express interest in participating). We also did not clarify the motivations for study participation (eg, strong views in favor of or against telehealth). A wide range of health professional disciplines involved in autism assessments was represented; however, there were comparatively fewer medically trained participants. Together, participants worked across different settings and types of services; however, we did not purposively recruit participants based on each service that may conduct autism diagnostic assessments (eg, the criminal justice system). All participants were based in England, which may have affected the generalizability of the findings to other countries.

### Conclusions

This is one of the first studies to explore, in-depth, health professionals’ views on conducting autism diagnostic assessments via telehealth in England since the onset of the COVID-19 pandemic. The study participants represented 7 clinical disciplines and conducted diagnostic assessments with children, adolescents, and adults across most regions of the country. Together, participants were enthusiastic about many ways in which telehealth can be efficient, flexible, and limit costs, with clear examples of innovation. However, it was also evident that some patients may wait for a disproportionately long time for assessment as telehealth is not deemed appropriate, given their clinical presentation, risk issues, or digital poverty. Views differed regarding the degree to which solely using telehealth is a sufficiently valid and reliable way of assessing autism and sharing diagnostic conclusions. Further studies are needed to establish what best practice telehealth autism diagnostic assessments should comprise, alongside research that focuses on reducing health care disparities and enhancing professionals’ skills and confidence in working in this way. In addition, the development of telehealth service provision should ideally incorporate stakeholder engagement and collaboration.

## Appendix

Multimedia Appendix 1 Supplementary information.

Multimedia Appendix 2 Theme 1 and indicative participant points.

Multimedia Appendix 3 Theme 2 and indicative participant points.

Multimedia Appendix 4 Theme 3 and indicative participant points.

Multimedia Appendix 5 Theme 4 and indicative participant points.

Multimedia Appendix 6 Theme 5 and indicative participant points.

Multimedia Appendix 7 Theme 6 and indicative participant points.

Multimedia Appendix 8 Theme 7 and indicative participant points.

## Abbreviations

ADOS: Autism Diagnostic Observation Schedule
MDT: multidisciplinary team
NHS: National Health Service
