# Supplementary material for: Telehealth Autism Diagnostic Assessments With Children, Young People, and Adults: Qualitative Interview Study With England-Wide Multidisciplinary Health Professionals
Source: JMIR Ment Health. 2022 Jul 20;9(7):e37901. doi: 10.2196/37901 (PMC9302612; doi:10.2196/37901)
Supplement: Multimedia Appendix 1 [file mental_v9i7e37901_app1.docx]

TOPIC GUIDE

Title of study

Assessment and diagnosis of autism spectrum disorders remotely: The ASSESS 2 Study

Interview

Demographic questions:

- I would like to ask you a few questions about your core profession, work setting and experience of working with people with autism.
- What is your core profession?
- What country do you work in?
- What setting do you currently work in?
  - Prompts:
    - National Health Service, independent practice or both
    - As part of a team, or sole practitioner
    - Does the service / setting also see people with intellectual disabilities?
- Which age range best describes the people you work with?
  - Child and young people, adults (including older adults), or lifespan
- For how many years have you worked with autistic people, for the majority of your professional time?
  - Do you work with other clinical populations as well?

Context:

- For context, please could you tell me a bit about how you / your service conducted diagnostic assessments prior to the pandemic?
  - Prompts:
    - What types of standardised measures were used?
    - Were any components of the assessment conducted remotely?
    - How long did the assessment take?

Perceptions of working remotely:

- Have you undertaken any remote diagnostic assessments?
  - Prompts:
    - Approximately how many (e.g. 10, 50, 100)?
    - What proportion of the diagnostic assessments you have conducted since March 2020 have been:
      - a) in person
      - b) remote
      - c) blended (i.e. incorporating in person and remote methods)?
    - What has the assessment comprised?
    - What methods of telehealth have you been using (e.g. Teams, FaceTime, telephone)?
    - Do you do the entire assessment remotely, or only part of this?
    - Is the assessment longer, shorter than prior to the pandemic?
- What is your view about remote assessments?
  - Prompts:
    - What do you like / dislike about this way of working?
    - In what way is this easier / more difficult than traditional in person assessments?
    - Is there anything that you do differently in advance of an assessment via telehealth, compared to in person assessments? or anything that you do differently in terms of feeding back to the person?

Barriers and facilitators to conducting remote diagnostic assessments:

- In your opinion, are there any ways in which working remotely makes components of an assessment easier?
- In your opinion, are there any barriers to:

1. remote working; and
2. reaching a diagnostic conclusion?
   - Prompts:
     - Does conducting an assessment remotely impact your confidence to reach a diagnostic conclusion?
     - Is it more difficult to assess and diagnose some symptoms / difficulties / conditions remotely?
     - Why do you think this is?
     - What factors make it difficult to reach a diagnostic conclusion via a remote assessment?
     - In your opinion, is there anything that can mitigate these barriers?

- Have there been any times when you have not been able to reach a diagnostic conclusion (when working remotely)?
  - Prompts:
    - Has this happened more or less often than in person appointments?
- What is it like telling someone that they (or their child):

a) do meet diagnostic criteria for autism, remotely, rather than in person?

b) do not meet diagnostic criteria for autism, remotely, rather than in person?

- - Prompts:
    - Does this seem different compared to in person working?
- Has working remotely impacted on how many assessments your service can do, i.e. more or less than when only working in person?
- Has the DNA [do not attend] rate changed since starting remote assessments?

Feedback from service users

- What feedback, if any, have you had from your patients or their families, about the advantages and limitations of remote diagnostic assessments?

Training and supervision

- What additional training, if any, do you think would be helpful for professionals doing diagnostic assessments remotely?
  - Have you received any additional training or support with regards to working remotely?
- Do you think there are any additional considerations for clinical supervision?
  - Prompts:
    - For supervisees and/or supervisors?

Improving service delivery:

- In what ways do you think we could improve the quality of remote autism assessments?
- What do you think a remote diagnostic assessment should ideally comprise?
  - Prompts:
    - What methods?
- Who (which configuration of health professionals) do you think should ideally be involved in the assessment?
- What innovations or new tools / methods do you think could help you with assessment and diagnosis under the current lockdown circumstances?
- After social distancing restrictions have been relaxed, will you continue to work in this way or would you prefer to meet people in person?
  - Prompts:
    - What would influence your decision-making about whether to see someone in person or remotely?
    - Confirm eligibility for online assessment

Post-diagnostic support:

- Are you involved in offering post-diagnostic support?
  - Prompts:
    - What does this comprise?
    - How is this different to before the pandemic?
- How do you think provision of remote post-diagnostic support could be improved?

COREQ checklist [23]

| **Topic** | **Item number** | **Reported on page no.** |
| --- | --- | --- |
| Domain 1: Research team and reflexivity | | |
| *Personal characteristics* | | |
| Interviewer/facilitator | 1 | Page 9 |
| Credentials | 2 | Page 9 |
| Occupation | 3 | Page 9 |
| Gender | 4 | Page 9 |
| Experience and training | 5 | Page 9 |
| *Relationship with participants* | | |
| Relationship established | 6 | Page 9 |
| Participant knowledge of the interviewer | 7 | Page 9 |
| Interviewer characteristics | 8 | Page 9 |
| Domain 2: Study design | | |
| *Theoretical framework* | | |
| Methodological orientation and theory | 9 | Page 6 |
| Sampling | 10 | Page 6 |
| Method of approach | 11 | Page 6 |
| Sample size | 12 | Page 7 |
| Non-participation | 13 | Page 7 |
| *Setting* | | |
| Setting of data collection | 14 | Page 9 |
| Presence of non-participants | 15 | Page 9 |
| Description of sample | 16 | Page 7-8 |
| *Data collection* | | |
| Interview guide | 17 | Page 8 |
| Repeat interviews | 18 | Page 6 |
| Audio/visual recording | 19 | Page 9 |
| Field notes | 20 | Page 9 |
| Duration | 21 | Page 9 |
| Data saturation | 22 | Page 6 |
| Transcripts returned | 23 | Page 10 |
| Domain 3: Analysis and findings | | |
| *Data analysis* | | |
| Number of data coders | 24 | Page 10 |
| Description of the coding tree | 25 | Page 12 |
| Derivation of themes | 26 | Page 9-10 |
| Software | 27 | Page 10 |
| Participant checking | 28 | Page 10 |
| *Reporting* | | |
| Quotations presented | 29 | Page 13-28 |
| Data and findings consistent | 30 | Page 12-28 |
| Clarity of major themes | 31 | Page 13-28 |
| Clarity of minor themes | 32 | Page 13-28 |
