# Supplementary figures and images for: Telehealth Autism Diagnostic Assessments With Children, Young People, and Adults: Qualitative Interview Study With England-Wide Multidisciplinary Health Professionals
Source: JMIR Ment Health. 2022 Jul 20;9(7):e37901. doi: 10.2196/37901 (PMC9302612; doi:10.2196/37901)

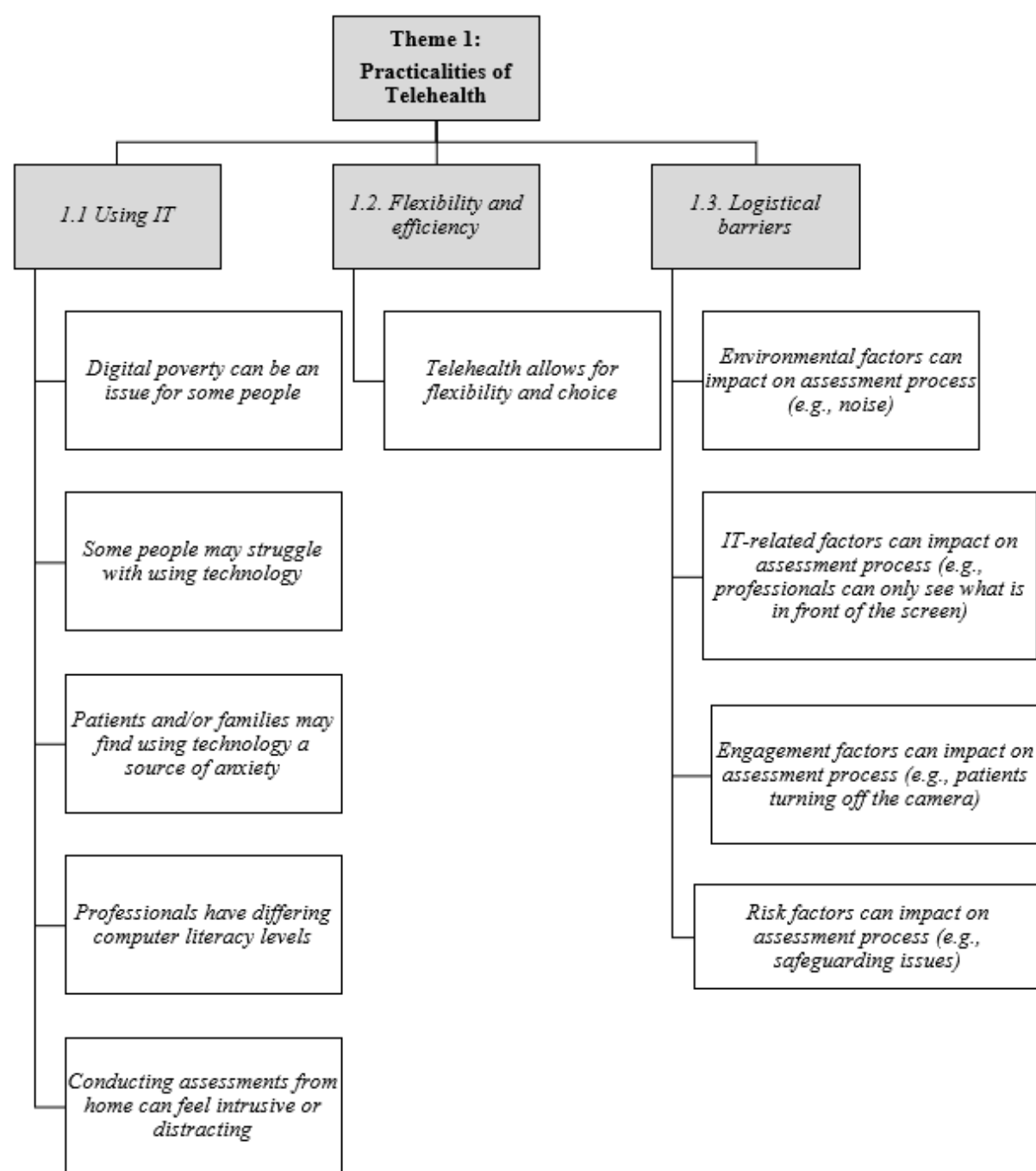

Supplement: Multimedia Appendix 2 [file mental_v9i7e37901_app2.pdf]

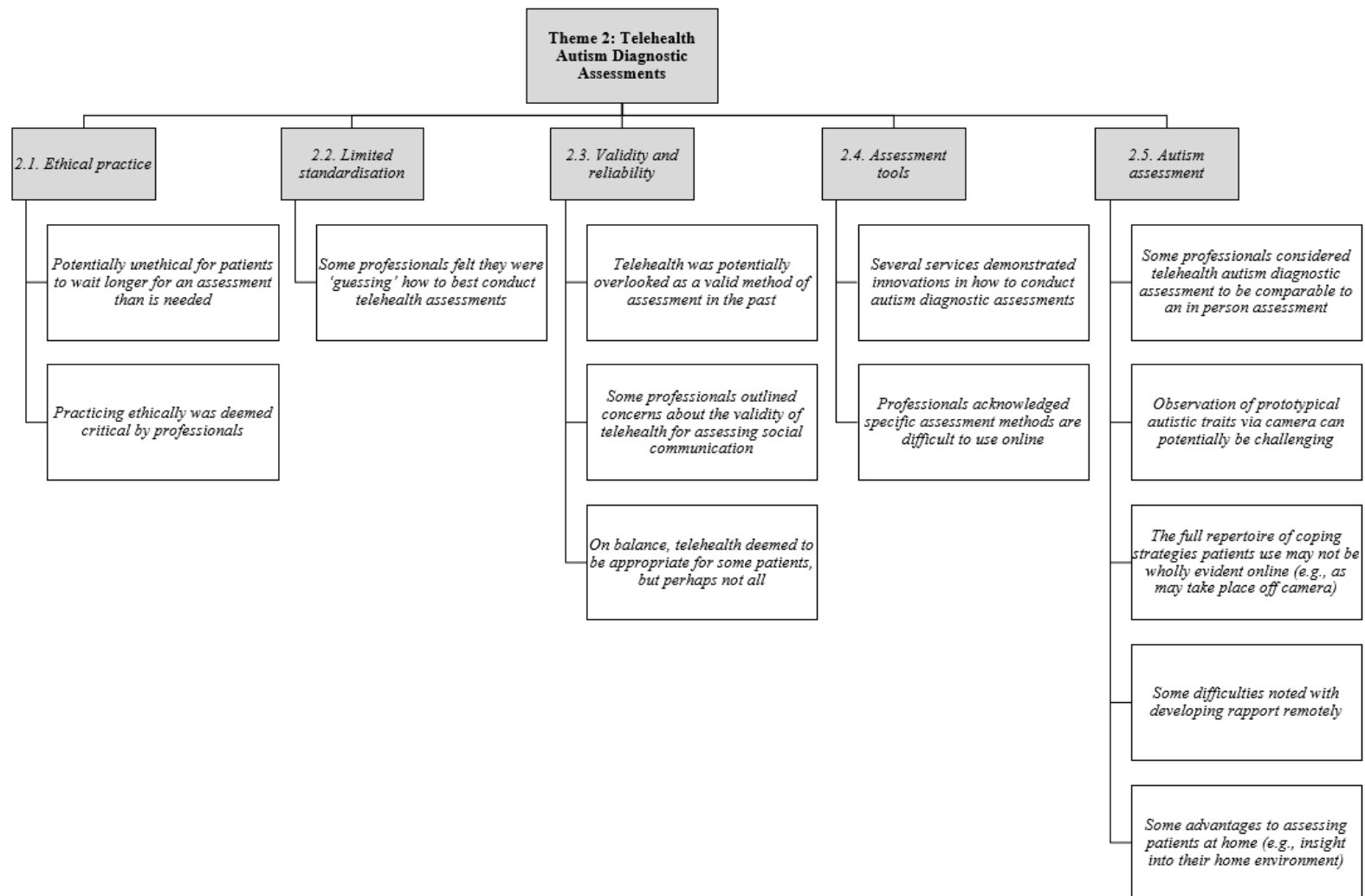

Supplement: Multimedia Appendix 3 [file mental_v9i7e37901_app3.pdf]

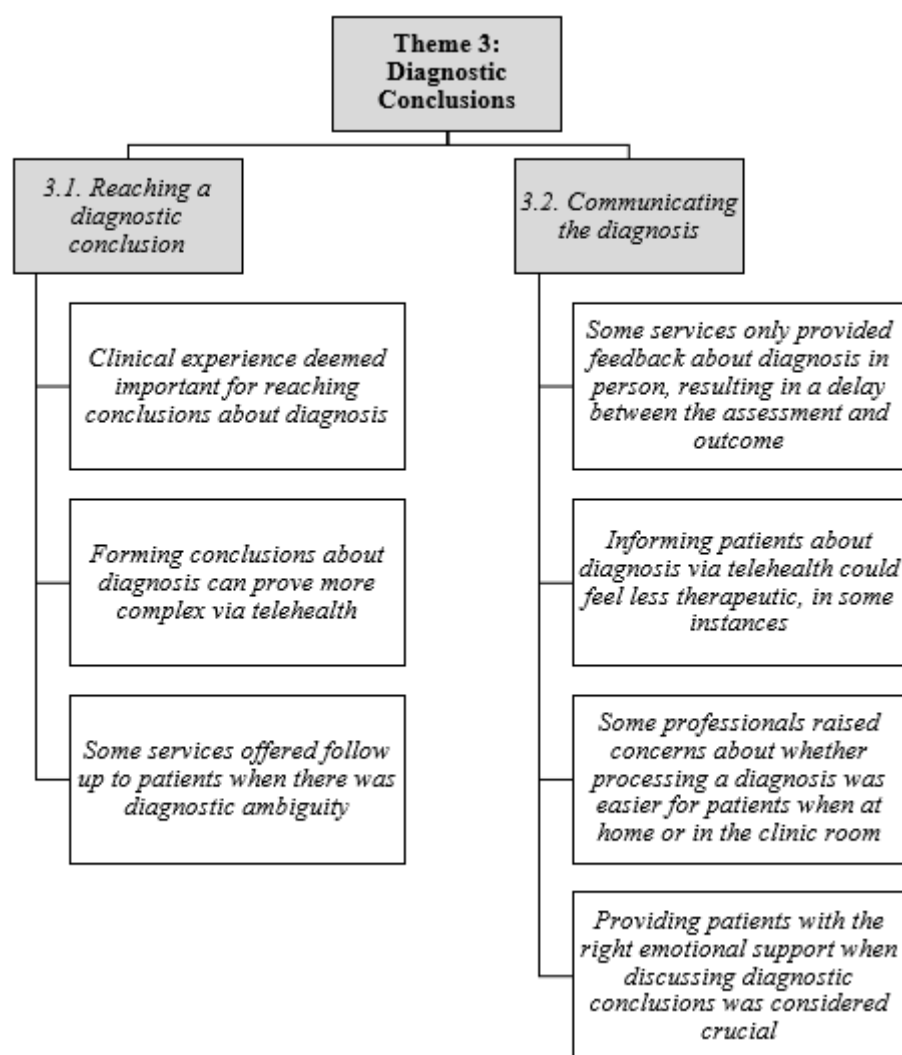

Supplement: Multimedia Appendix 4 [file mental_v9i7e37901_app4.pdf]

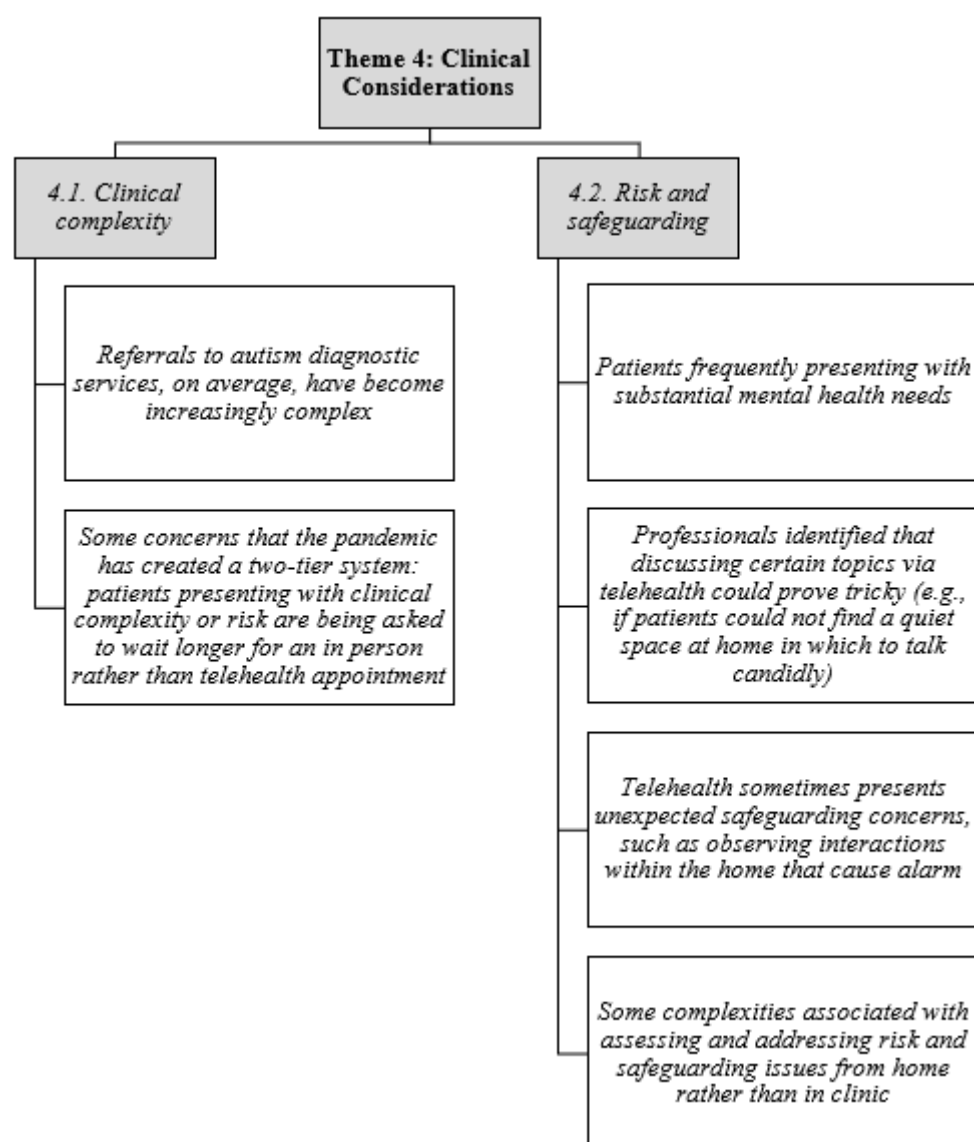

Supplement: Multimedia Appendix 5 [file mental_v9i7e37901_app5.pdf]

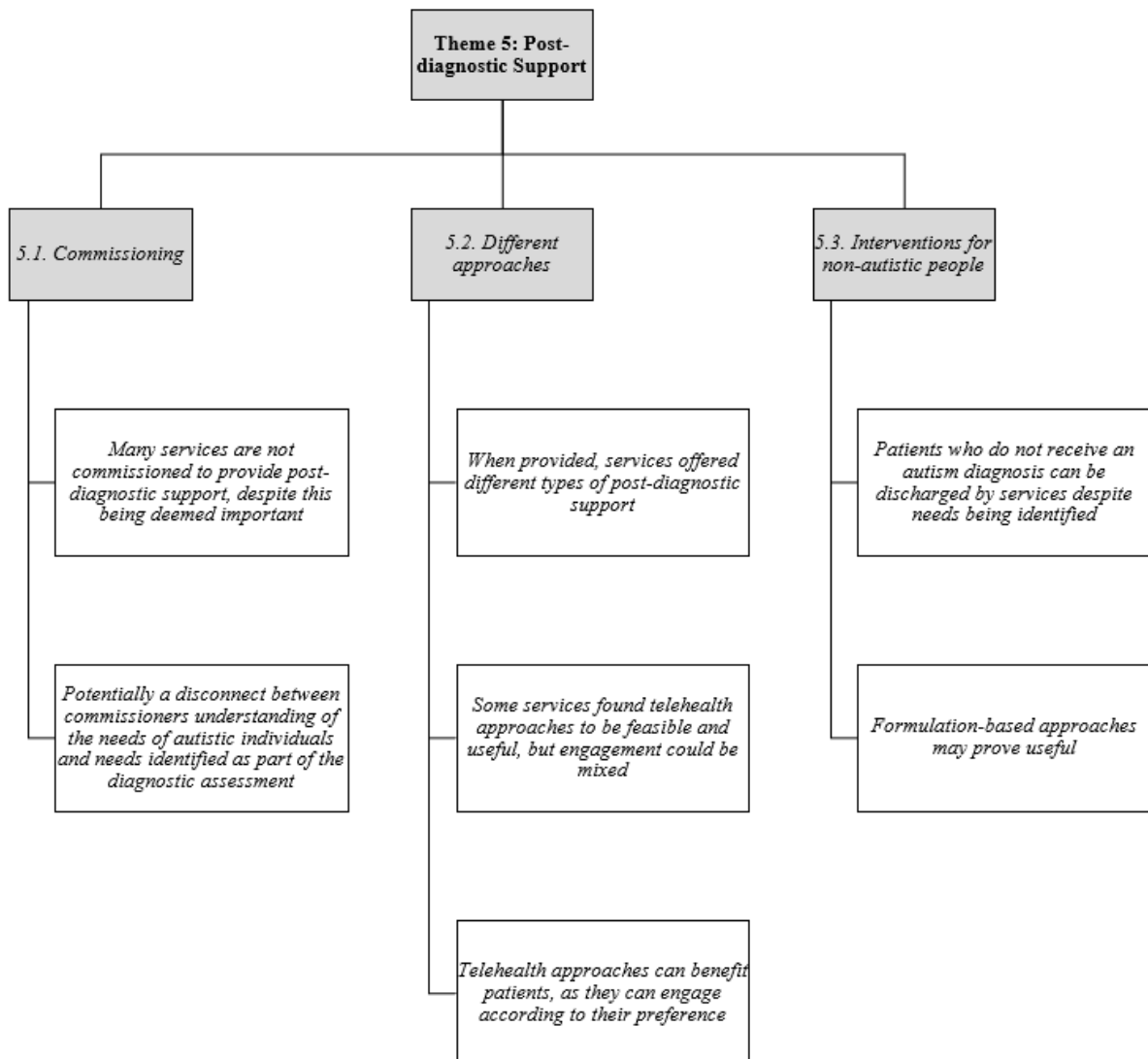

Supplement: Multimedia Appendix 6 [file mental_v9i7e37901_app6.pdf]

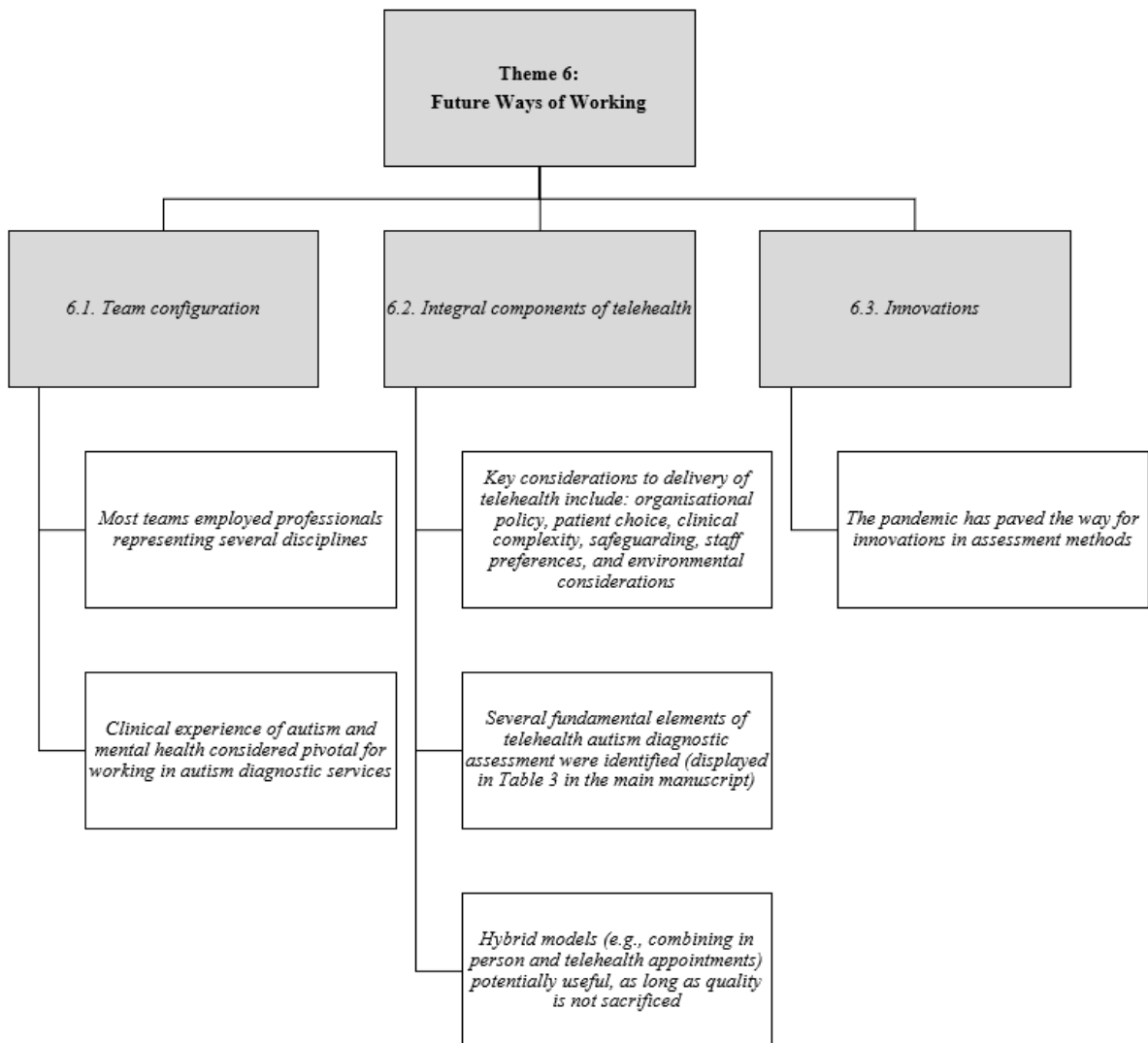

Supplement: Multimedia Appendix 7 [file mental_v9i7e37901_app7.pdf]

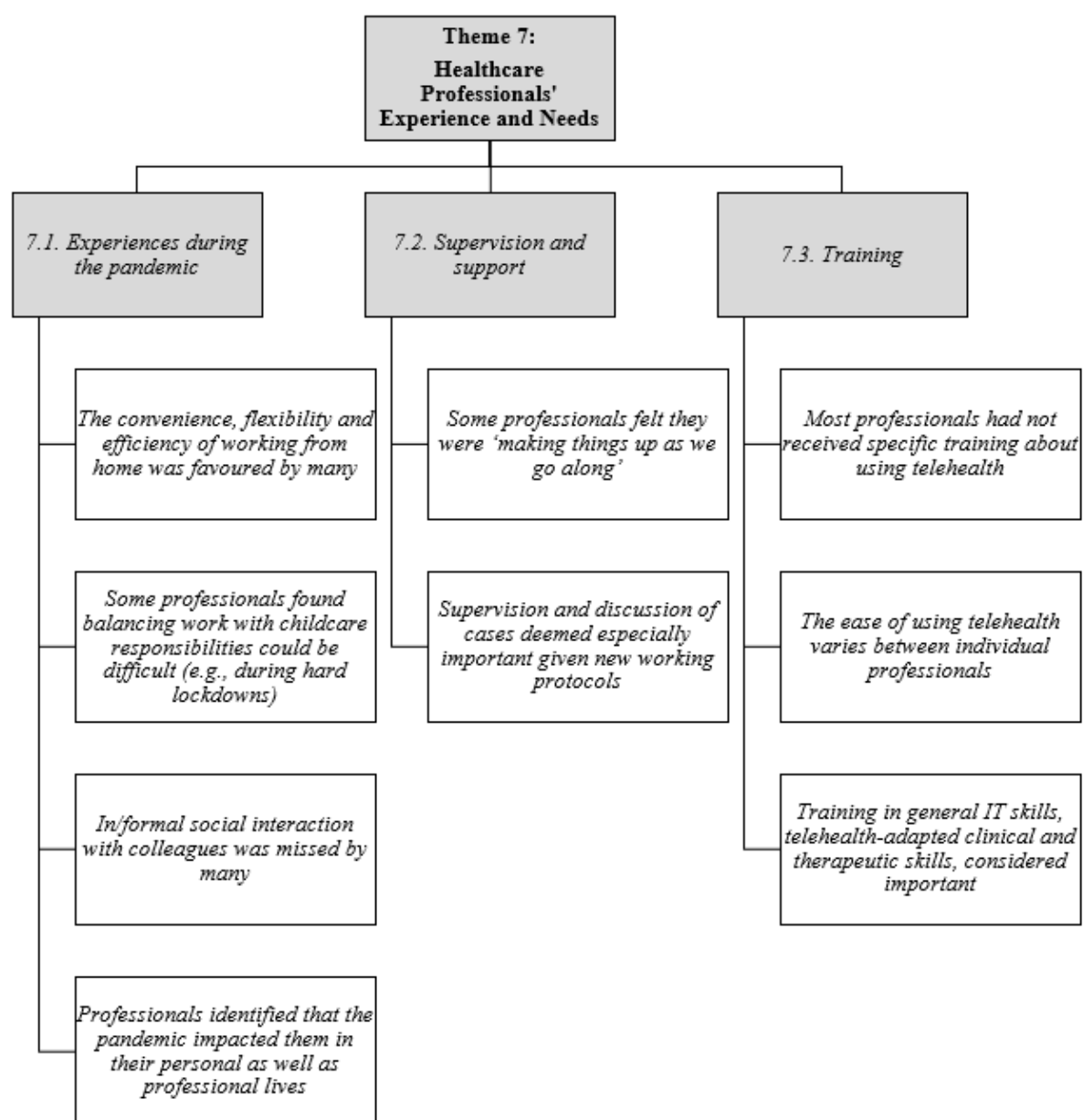

Supplement: Multimedia Appendix 8 [file mental_v9i7e37901_app8.pdf]
